# The Pathophysiology, Prognosis and Treatment of Hypertension in Females from Pregnancy to Post-menopause: A Review

**DOI:** 10.1007/s11897-024-00672-y

**Published:** 2024-06-11

**Authors:** Simeng Li, Isabella Tan, Emily Atkins, Aletta E Schutte, Sonali R Gnanenthiran

**Affiliations:** 1 School of Medicine, The University of Notre Dame Australia, Sydney, NSW 2010 Australia; 2 The George Institute for Global Health, University of NSW, Barangaroo, NSW 2000 Australia; 3 Department of Cardiology, Concord Repatriation Hospital, Concord, NSW 2139 Australia

**Keywords:** Hypertension, Blood Pressure, Female, Gestational, Pregnancy, Menopause

## Abstract

**Purpose of Review:**

We summarise the physiological changes and risk factors for hypertension in females, potential sex-specific management approaches, and long-term prognosis.

**Key Findings:**

Pregnancy and menopause are two key phases of the life cycle where females undergo significant biological and physical changes, making them more prone to developing hypertension. Gestational hypertension occurs from changes in maternal cardiac output, kidney function, metabolism, or placental vasculature, with one in ten experiencing pregnancy complications such as intrauterine growth restriction and delivery complications such as premature birth. Post-menopausal hypertension occurs as the protective effects of oestrogen are reduced and the sympathetic nervous system becomes over-activated with ageing. Increasing evidence suggests that post-menopausal females with high blood pressure (BP) experience greater risk of cardiovascular events at lower BP thresholds, and greater vulnerability to treatment-related adverse effects.

**Summary:**

Hypertension is a key risk factor for cardiovascular disease in females. Current BP treatment guidelines and recommendations are similar for both sexes, without addressing sex-specific factors. Future investigations into ideal diagnostic thresholds, BP control targets and treatment regimens in females are needed.

## Introduction

High blood pressure (BP), also known as hypertension, is a leading modifiable risk factor for congestive cardiac failure (CCF), affecting those with both preserved and reduced ejection fraction [1]. High BP is underdiagnosed and undertreated, with suboptimal control rates which are as low as 23% among females [2, 3]. The prevalence of hypertension in CCF patients is higher among females, with high BP associated with a three-fold risk of CCF in females compared to two-fold in males [4]. Treating hypertension could dramatically reduce the risk of CCF and other cardiovascular disease.

Although sex specific differences are not completely understood, there is evolving evidence about the fundamental differences in the pathophysiology of high BP in females compared to males [5–7]. Hypertension is a leading cause of morbidity and mortality in both males and females. Sex differences can influence the occurrence, severity, and pathogenesis of hypertension. These differences arise from differences in biological and behavioural factors associated with sex, such as hormone changes, and lifestyle risk factors [2]. Females are at higher risk of developing hypertension at certain stages of life, particularly during pregnancy and post-menopause. Understanding these stages is imperative for regulating BP and improving clinical outcomes in females. Apart from unique biological risk factors compared to men, females are more likely to be diagnosed with hypertension and are more likely to receive treatment, but appear to be less responsive to antihypertensive treatments [3]. Existing guidelines also do not target sex-specific factors when advising antihypertensive therapy. Despite a deeper understanding of physiological changes, there is still a gap translating this knowledge into clinical practice.

Our aim is to report the available evidence on the epidemiology, natural history, and efficacy and safety of treatment for hypertension during the two periods of physiological change that correspond to higher risk of hypertension in females: during pregnancy and post-menopause.

## Epidemiology

Hypertension is the leading modifiable contributor to mortality and morbidity globally [8]. In 2019, 22% of people aged ≥ 15 years reported having hypertension [4]. The prevalence of hypertension does, however, vary with sex after puberty. In general, the prevalence of hypertension is higher in males than females from adolescence to menopause [4, 7]. Shen et al. examined BP data from the Bogalusa Heart Study and found no sex differences in those aged 5 to 14 years. However, both systolic and diastolic BP starts to rise in males more than females from the age of 15 years onwards [7]. Systolic BP is on average 10 mmHg higher in males compared with females by the age of 18 years, a difference which slightly increases over time up to 30 years [9]. Although premenopausal females have a much lower BP than age-matched men, the pattern begins to reverse from the third decade of life where there is an increase in systolic, diastolic and mean BP observed in females [10].

Females experience sex-specific biological processes that increase their hypertension risk. The two most important stages where they are at highest risk are pregnancy and menopause. During normal pregnancy, BP starts to drop during the first trimester and will return to pre-pregnancy levels by term. However, hypertension complicates up to 5–16% of pregnancies worldwide [11]. The total incidence of hypertension in pregnancy has increased from 16.3 million to 18.1 million globally, with a total increase of 10.9% from 1990 to 2019 [12]. Specifically, the prevalence of hypertension during pregnancy is 16% in Europe [12], with prevalence varying between ethnic groups. A study including 150 million females from the National Hospital Discharge Survey demonstrated that black females were twice as likely to develop hypertension during pregnancy as white females (1.2% vs. 0.5%), and have a higher tendency to have earlier disease onset and poorer control of hypertension [13].

A second period where females experience a high prevalence of hypertension is during the menopause transition and post-menopause. The risk of hypertension is also dependent on the age at which a woman experiences menopause or menopausal symptoms. Menopause (cessation of menstrual period) is normally experienced by females aged between 45 and 55 years. An increase in systolic BP is observed in one-third of females with premature menopause (defined as cessation of menstrual periods in females aged < 40 years old) [14]. A study in females aged 44 to 56 years conducted by Son et al. found the prevalence of hypertension in late menopausal transition (defined as two skipped menstrual cycles and an interval of amenorrhea ≥ 60 days) was almost 6 times higher than those in early menopausal transition (defined as two or more menstrual cycles of ≥ 7 days difference in cycle length ; 6.1% vs. 1.4%), with BP significantly higher in later phase of menopausal transition than in early phase [15]. Following menopause, it is estimated that up to 41% of females develop hypertension, almost double the prevalence of premenopausal females [16]. Hence, hormonal changes and other physiological changes during menopause may contribute to the development of hypertension. After menopause, there is a marked rise in females’ BP levels, often higher than those observed in males [2].

### Gestational Hypertension

Hypertension in pregnancy may be chronic (predating pregnancy or diagnosed before 20 weeks of gestation) or de novo (hypertension that appears after 20 weeks of gestation) [17]. Two types of de novo hypertension in pregnancy are gestational hypertension (GH; where office systolic BP ≥140 mmHg and/or diastolic BP ≥90 mmHg on at least two occasions, but typically resolves within 42 days postpartum), and pre-eclampsia (where office systolic BP ≥140 mmHg and/or diastolic BP 90 mmHg) and is accompanied by other signs such as proteinuria or organ dysfunction [18]. In this review paper, we will focus on GH.

### Pathophysiology

**Fig. 1:**
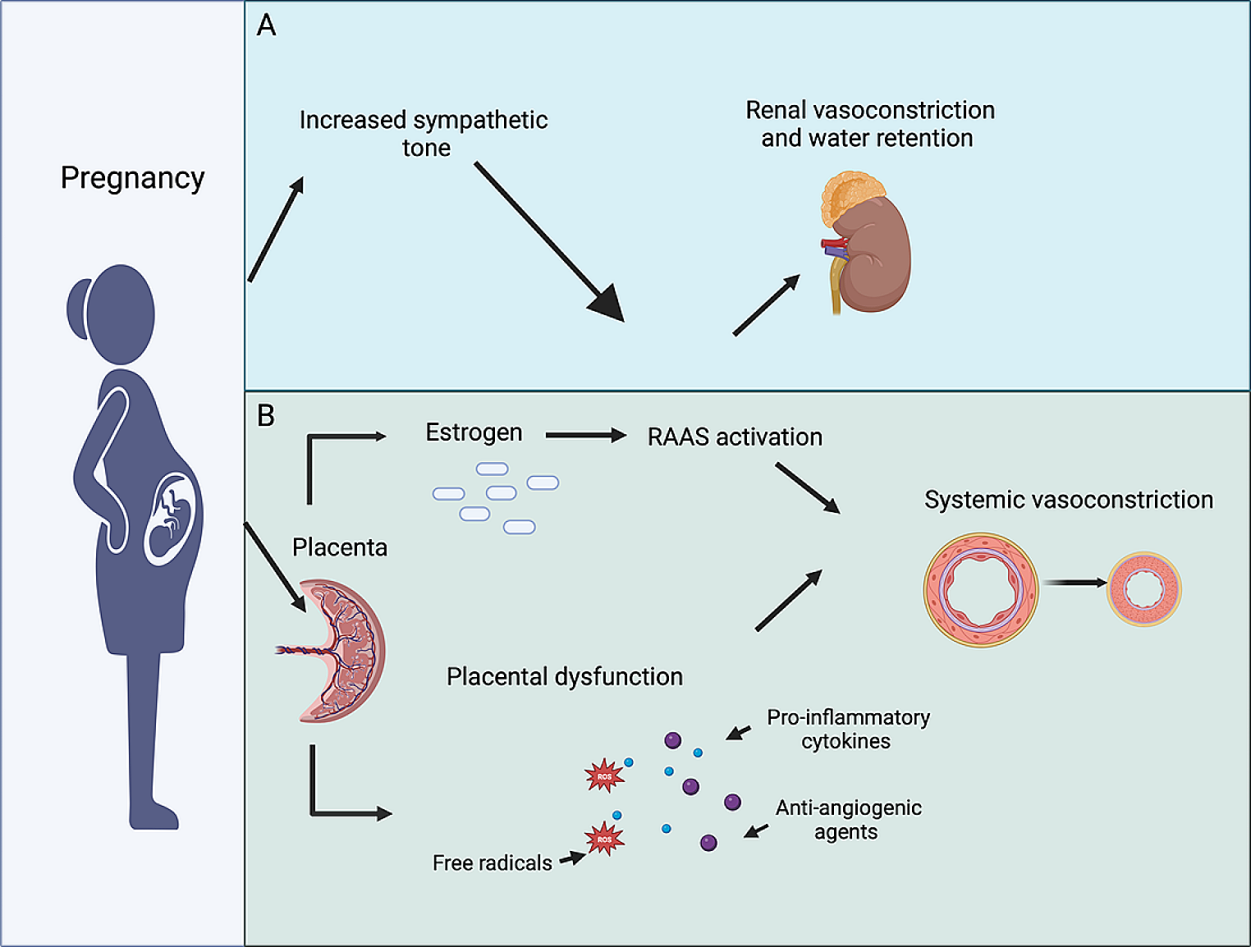
Physiological changes associated with pregnancy that predispose to increased blood pressure

One explanation of the development of GH is that it is the result of major physiological changes to meet the increased maternal and foetal demands during pregnancy (Fig. 1). Firstly, cardiac output increases in the first trimester of pregnancy and continues to increase into the second trimester and beyond by approximately 50% [19]. Secondly, there are changes in kidney function caused by increased oestrogen production, subsequently leading to an increase in plasma renin concentrations until 28 to 30 weeks of gestation (4.7 ng/mL/h vs. 15.7 ng/mL/h; *p* < 0.001) [20]. The rise in plasma renin concentration leads to increased activation of the renin-angiotensin-aldosterone system and subsequent salt and water retention, resulting in increased blood volume and BP [21]. Thirdly, the metabolism changes considerably during pregnancy. Pregnancy produces diabetogenic effects on metabolism through progressive insulin resistance that begins near mid-pregnancy [22]. During pregnancy, insulin resistance, combined with weight gain and dyslipidaemia, leads to a marked increase in sympathetic activity, visceral obesity, endothelial dysfunction, and increased activation of the renin-angiotensin system. This results in vasoconstriction, increased intravascular fluid and reduced vasodilation, all factors predisposing to hypertension [23–25].

### Treatment

Despite variations in recommendations and guidelines, it is crucial to monitor and control BP during pregnancy [26, 27]. The threshold for anti-hypertensive medication commencement is > 140/90 mmHg for females with GH [18]. The target BP should be < 140/90 mmHg for all hypertensive pregnant females [26]. Management methods depend on BP and maternal and foetal risk factors. Although treatment is necessary to prevent maternal and foetal morbidity and mortality, there is no controlled universal treatment protocol recommended. Clinicians need to consider the urgency and timing of starting treatment, as well as the choice of medication. This review will focus on pharmacological management in GH rather than lifestyle modifications, which have shown negligible effects [28, 29]. First line therapy for treatment of GH is generally labetalol and methyldopa [18].

#### Beta-blockers

Beta-blockers are the first-line medication during pregnancy and lactation. Labetalol is considered the first-line choice for chronic hypertension in pregnancy, especially severe hypertension. A meta-analysis collectively concluded that compared with placebo, oral labetalol 200 mg reduced severe hypertension (OR 0.33, 95% CI 0.20–0.52), pre-eclampsia (OR 0.73, 95% CI 0.54–0.99) and foetal death (OR 0.54, 95% CI 0.30–0.98) in pregnant females with mild to moderate hypertension [30]. While the efficacy of labetalol is well supported, there is not much evidence to support its safety. A randomised controlled trial by Easterling et al. comparing the safety and efficacy of labetalol 200 mg and methyldopa 100 mg in pregnant females with severe hypertension (systolic BP ≥160 mmHg or diastolic BP ≥110 mmHg) at least 28 weeks of gestation showed no difference in BP control between the labetalol and methyldopa groups (84% vs. 77%, *p* = 0.80). However, the use of labetalol was associated with a higher incidence of adverse maternal outcomes including hypotension (BP < 120/<70 mmHg), foetal distress, or severe headache (*p* = 0.03) [30]. Lardoux et al. conducted a trial to compare the safety and efficacy of labetalol 614±47.8 mg/day vs. atenolol 144.6±47.8 mg/day in 65 females with GH (BP > 140/>90 mmHg, 23.4 weeks of gestation) [31]. Although there was no significant difference between the two drugs in terms of BP lowering (-26.7±-6.1/-15.3±-5.3 mmHg vs. -20.7±-0.1/-11.018.9±-2.0 mmHg, *p* > 0.001) in this study, birth weight was significantly higher with labetalol than with atenolol (3289 vs. 2750, *p* < 0.001). In addition, the reduction in heart rate was also greater with atenolol than with labetalol (93.8±9.3 to 75.4±9.7/min vs. 92.3±11.1 to 86.5±6.3/min, *p* < 0.001) [31]. Despite their efficacy, β-blockers can cross the placenta and may cause neonatal bradycardia and hypoglycaemia [32].

#### Methyldopa

Methyldopa is a centrally acting α_2_-adrenergic receptor agonist, which decreases catecholamine release and thus central sympathetic outflow. By reducing sympathetic outflow, methyldopa exerts its effects in the periphery, inhibiting vasoconstriction and reducing vascular resistance. Methyldopa use in pregnancy has been well established in clinical practice. Previous studies have demonstrated the efficacy of methyldopa in the treatment of mild to moderate hypertension (140–170/90–110 mmHg) [33, 34]. A trial conducted by Redman et al., showed a much lower rate of progression to severe hypertension (systolic BP > 170 mmHg, or diastolic BP > 110 mmHg on two occasions more than 4 h apart) in females with moderate hypertension in pregnancy (BP > 140/>90 mmHg) treated with methyldopa 0.75 to 1.0 g/day in the second and third trimester (7.7% vs. 16.8%, *p* < 0.04) [34]. Another trial by Rezk et al. also reported a much lower rate of severe hypertension in the study group treated with methyldopa 1–2 g/day (23.2% vs. 53.1%, OR 0.27, 95%CI − 0.17-0.43, *p* < 0.001), and a lower rate of placental abruption (6.1% vs. 23.5%, OR 0.21, 95%CI 0.10–0.44, *p* < 0.001) [35]. Salama et al. also showed consistent findings, with pregnant females with mild to moderate hypertension treated with methyldopa 1–2 g/day at lower risk of severe hypertension (22.9% vs. 53.6%, OR 0.26, 95%CI 0.16–0.41, *p* < 0.001) [36]. A meta-analysis examining maternal and foetal safety outcomes showed no difference in the risk of neonatal adverse effects including risk of preterm birth and long-term growth restriction, when comparing methyldopa with other antihypertensives (adjusted RR 0.77, 95% CI 0.52 to 1.14; 22 trials, *n* = 1791), including calcium channel blockers (adjusted RR 0.90, 95%CI 0.52–1.57; nine trials, *n* = 700) or with beta blockers (adjusted RR 1.23, 95%CI 0.81–1.88; 19 trials, *n* = 1625) [37]. Although methyldopa has been an acceptable first-line drug, depression is a known side effect [38]. Nayak et al. examined 100 females with GH treated with methyldopa and without endocrinological or mental diseases history [38], and found that 77.8% developed postpartum depression (OR 6.45, *p* = 0.026) [38]. It has been suggested that females with a history of depression should therefore avoid methyldopa.

#### Calcium Channel Blockers

Calcium channel blockers are commonly used to treat hypertension during pregnancy and lactation. Of this class, nifedipine has the most available data to demonstrate its safety and efficacy in pregnancy [39]. In a study of pregnant females with mild-to-moderate hypertension, nifedipine 20–40 mg/day was effective in reducing the development of severe hypertension compared to placebo (22.5% vs. 53.5%, *p* < 0.001) and pre-eclampsia (26.5% vs. 48.8%, *p* < 0.001) [36]. Several studies have compared the safety and efficacy of nifedipine with other drugs [30, 39]. A clinical trial comparing the efficacy and safety of nifedipine 10 mg and methyldopa 200 mg in pregnant females with severe hypertension (BP ≥180/110) showed better BP control with nifedipine (84% vs. 76%, 95%CI 0.8–13.5), and a lower incidence of maternal adverse events (hypotension BP < 120/<70 mmHg and foetal distress, *p* = 0.03) [30]. The Pregnancy and Chronic Hypertension: Nifedipine Versus Labetalol as Antihypertensive Treatment study showed no significant difference in BP control (mean difference systolic: 1.2 mmHg [-4.9 to 7.2 mmHg], diastolic: 3.3 mmHg [-0.6 to 7.3 mmHg], RR 1.01, 95%CI 0.71–1.81), adverse maternal events (RR 1.78, 95%CI 0.84–3.77), and mean birth weight (2730 g vs. 2960 g) between groups of females with GH treated with labetalol (200–1800 mg/day) and nifedipine (20–80 mg/day) [39]. The clinical benefits of nifedipine in the management of BP in pregnancy are consistent and comparable to other antihypertensives. However, there is a potential interaction between nifedipine and magnesium sulphate to control BP in pregnant females including profound hypotensive response [40, 41]. A few case reports also showed interactions may lead to neuromuscular blockade and myocardial depression in females with pregnancy induced hypertension (*p* < 0.05) [40–42]. Overall, this suggests caution should be taken when these therapies are used concurrently. Given the lack of available data, large scale studies are required to investigate the effects of the combination of magnesium sulphate and nifedipine.

#### Other Drugs

Drugs that are contraindicated in pregnancy and lactation, such as angiotensin-converting enzyme (ACE) inhibitors, aldosterone receptor blockers (ARBs) and direct renin inhibitors, should be strictly avoided as they may cause potential foetal toxicity [43]. For example, ACE inhibitors and ARBs are teratogenic, and can be associated with renal dysplasia, growth retardation, ossification disorder of skull, and lung hypoplasia [44]. A systematic review showed 48% of 118 foetuses exposed to ACE inhibitors, and 87% foetus exposed to ARBs developed complications related to these medications [44].

There are very limited trials to evaluate the safety and efficacy of hydralazine in pregnancy. In a meta-analysis of 21 trials (8 of which compared hydralazine with nifedipine and 5 with labetalol [44]), hydralazine was associated with more maternal side effects when compared to other commonly used agents including increased risk of maternal hypotension (RR 3.29, 95%CI 1.50–7.13; 13 trials); placental abruption (RR 4.17, 95%CI 1.19–14.28; 5 trials); adverse effects on foetal heart rate (RR 2.04, 95%CI 1.32–3.16); 12 trials), and lower Apgar scores (RR 2.70, 95%CI 1.27–5.88; 3 trials) [45]. Hydralazine should not be used as a first-line treatment for hypertension during pregnancy. The use of hydralazine in the third trimester is therefore generally not recommended unless the benefits outweigh the risks to the foetus and no other regimen achieves adequate BP management targets [43].

### Prognosis

Although GH usually resolves within 12 weeks postpartum, it can lead to maternal complications during pregnancy such as renal dysfunction, postpartum haemorrhage, disseminated intravascular coagulation, and even mortality. A prospective observational study of 112 females with pregnancy-induced hypertensive disorders found postpartum haemorrhage was the most frequent complication at delivery (31%), followed by placental abruption (19%), and acute renal failure (7%) [46]. 

Pregnancy-related hypertensive disorders can also lead to perinatal complications. The most frequent complication is intrauterine growth restriction (OR 2.16; 95%CI 1.10–4.24; *p* = 0.026) [47]. McCowan et al. observed that females with pregnancy-related hypertensive disorders had a 2.5-fold increase in the rate of small for gestational age babies (OR 2.9; 95%CI 1.6-5.0). Similarly, a prospective study involving 822 females with GH also found that GH led to a 2-time higher rate of small for gestational age babies (RR 2.30; 95%CI 1.85–2.84); and a 5-time higher rate in having pre-term deliveries (RR 3.52; 95%CI 2.79–4.45) [48]. Although the mechanism is not fully understood, it is hypothesised that hypertension during pregnancy disrupts the proper remodelling of spiral arteries and formation of the uteroplacental blood supply. This disruption leads to reduced oxygen and nutrient transfer to the developing foetus, ultimately resulting in intrauterine growth restriction.

Hypertension during pregnancy can have adverse implications on cardiovascular health later in life. The long-term prognosis of hypertension in pregnancy was evaluated as early as the 1960s by Adams and MacGillivray, who noted that females with a history of pregnancy-related hypertension were at an increased risk of developing hypertension later in life, even before onset of menopause (RR 1.41) [49]. Similar findings were observed in other studies [35, 50–54]. Marin et al. conducted a study (634 females with GH, 285 females with normotensive pregnancies, mean follow up 13.6 years) [52] which demonstrated that females with GH have higher prevalence of subsequent hypertension (45% vs. 14%, odds ratio [OR] 5.1, 95%CI 2.5–9.8, *p* < 0.001) [52]. In addition, evidence suggests an association between GH and future endothelial dysfunction, kidney damage, heart failure and coronary heart disease [55]. Females with hypertensive pregnancies were more likely to develop left ventricular hypertrophy in the year after delivery (OR 1.42, 95%CI 1.01–1.99, *p* = 0.05) [56], with a 4-fold increase in the incidence of future CCF (RR 4.19, 95% CI 2.09–8.38) [53], and a 2-fold increase in the incidence of coronary heart disease (RR 1.81, 95% CI 1.43–4.37) [53].

## Post-menopausal Hypertension

### Pathophysiology

Studies have confirmed that hypertension is twice as likely post menopause than in premenopausal females [14]. There may be multiple aetiologies and triggers for hypertension in post-menopausal females, with renin-angiotensin-aldosterone system dysregulation, sympathetic activation and declining oestrogen levels being key factors. Animal studies suggest that multiple mechanisms are likely involved in post-menopausal hypertension [57]. 

**Fig. 2:**
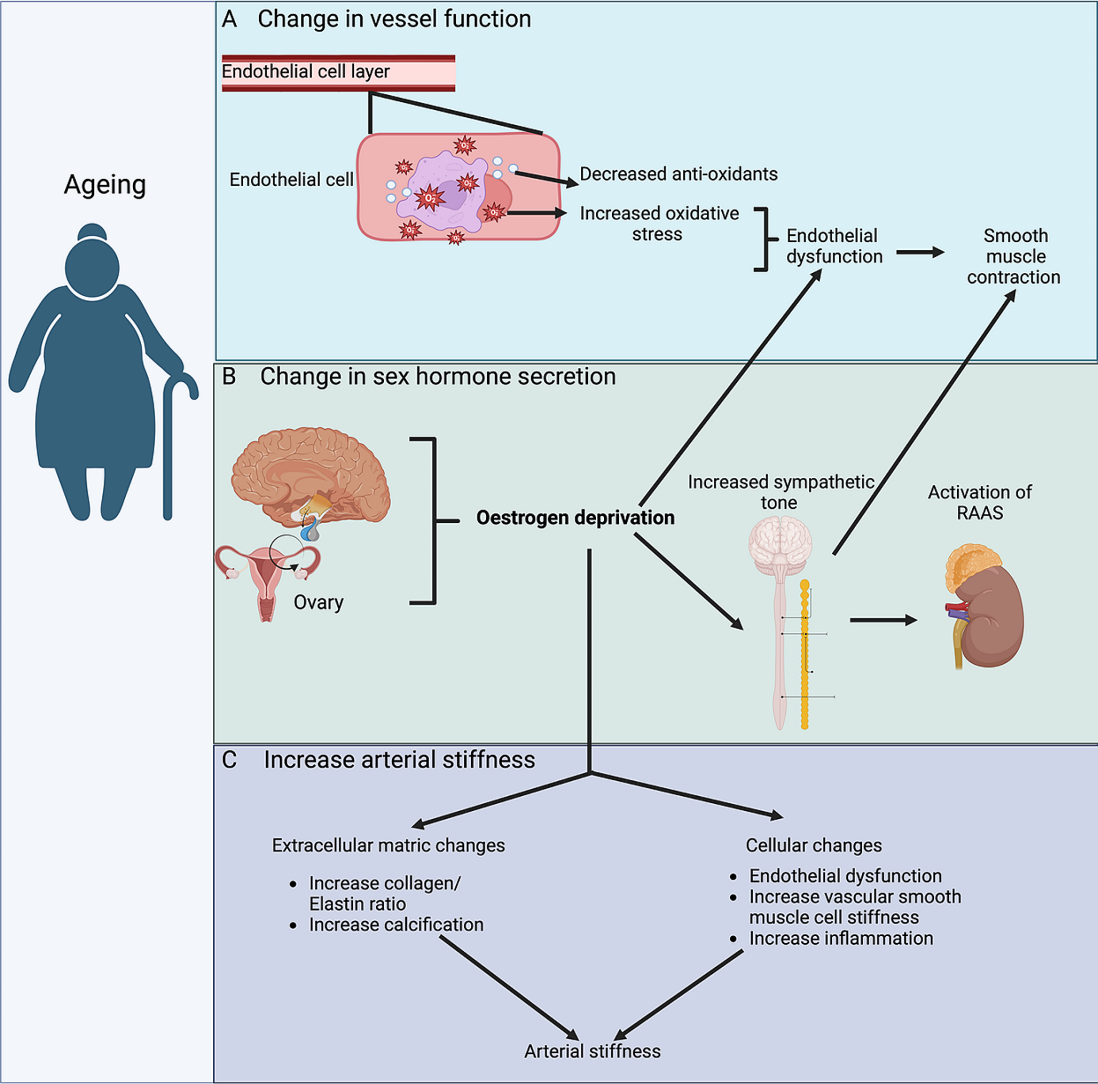
Pathophysiological changes associated with ageing that predispose to increased blood pressure in post-menopausal women

During the menopausal transition, females are subjected to age-related changes in vascular function and sex hormone production due to declining ovarian function, which increases susceptibility to hypertension more so than age-matched males (Fig. 2) [58]. Ageing alters the structure and function of the vascular system, resulting in progressive arterial stiffening and decreased vasodilatory capacity [58, 59]. During ageing, lifestyle and biological factors modulate vascular function, increasing vascular oxidative stress and reducing antioxidant defences [58, 60]. Increased vascular oxidative stress leads to progressive dysfunction of the endothelial cell layer of the vascular wall. Dysfunction of the endothelial cell layer results in an imbalance in the secretion of endothelium-derived substances [58]. This imbalance impairs arterial endothelium-dependent vasorelaxation, enhancing constriction and vascular remodelling, which increases peripheral vascular resistance and is involved in the development of hypertension [61].

Menopause-induced changes in sex hormones are associated with an increase in cardiovascular risk factors. The hormonal changes associated with menopause include a decline in plasma levels of oestrogen and a marked increase in follicle-stimulating hormone and luteinizing hormone. Oestrogens have cardioprotective effects via multiple pathways. Firstly, oestrogen increases nitric oxide bioavailability, decreases oxidative stress and inflammation, and inhibits protein kinase C and Rho-kinase-mediated vascular smooth muscle contraction [62]. Therefore, the combination of oestrogen deprivation and endothelial dysfunction generated by ageing increases vascular resistance. Secondly, oestrogen helps maintain and regulate autonomic balance by enhancing the heart’s vagal impact and decreasing its sympathetic activity [63]. Studies in post-menopausal females with oestrogen deficiency have shown changes in autonomic tone, such as decreased baroreflex sensitivity and decreased vagal tonic regulation, resulting in a dominance of sympathetic tone [64–66]. In addition, there are changes in the renin-angiotensin-aldosterone system, a key regulator of BP, in menopause. Post-menopausal females exhibit increased plasma renin activity [67, 68], which is thought to be a result of sympathetic dominance. In addition, sympathetic dominance also leads to a decrease in heart rate variability, which increases the risk of cardiovascular events in post-menopausal females [64].

In addition, the menopausal period can also be associated with the emergence of metabolic syndrome including accumulation of central adiposity, shifts to a more atherogenic lipid profile, and increased blood glucose level due to insulin resistance – all of which contribute to elevated cardiovascular risk [69]. Population-based cohort studies suggest that the BP increase during menopausal transition can largely be attributed to weight gain, obesity and ageing [70, 71].

### Treatment

Management approaches, therefore, need to consider targeting the multiple pathways involved in post-menopausal hypertension. Although the biological differences are clear in the pathogenesis of hypertension, there is no clear sex-specific treatment approach for hypertension. Evidence generally demonstrates comparable reductions in BP and incidence of cardiovascular events in both sexes across the major antihypertensive treatment classes of ACE inhibitors, ARBs, calcium channel blockers, diuretics or beta blockers [72]. Consideration is needed about whether a sex-specific treatment strategy is needed, as well as addressing the underrepresentation of females in BP trials, who generally constitute only 25-30% of participants [73].

#### Angiotensin-converting Enzyme Inhibitors

ACE inhibitors are a commonly used medication in the treatment of hypertension and may target menopause induced renin-angiotensin-aldosterone system dysregulation, although animal studies suggest that this is only one pathway involved [57]. Koch et al. compared the safety and efficacy of the ACE inhibitor moexipril 15 mg vs. placebo in 95 hypertensive post-menopausal females, and found that after 12 weeks of treatment, moexipril significantly reduced baseline systolic and diastolic BP compared to placebo (-12.2/ -9.9 mmHg vs. -1.6/ -4.3 mmHg, *p* < 0.001) [74]. Moexipril was well tolerated, with no significant difference between the moexipril and placebo groups [74]. A few studies compared the safety and efficacy of ACE inhibitors with other commonly used anti-hypertensive drugs such as atenolol and hydrochlorothiazide [75, 76]. For example, Stimpel et al. compared the efficacy and safety of moexipril 15 mg/day with hydrochlorothiazide 25 mg/day over 12 weeks in post-menopausal hypertensive females aged 42–72 years and found that moexipril achieved equivalent BP lowering to hydrochlorothiazide, but moexipril was better tolerated by post-menopausal females (*p* < 0.001) [75]. Stimpel et al. conducted a separate study comparing the safety and efficacy of moexipril 15 mg and atenolol 25 mg. After 12 weeks of treatment in post-menopausal females with mild to moderate hypertension, they found that moexipril and atenolol were comparable in their efficacy of lowering BP and that both drugs were well tolerated during the study period [77].

#### Angiotensin II Receptor Blockers

Blockade of the renin angiotensinogen system with an ARB is also a commonly used regimen for BP control, and may target renin-angiotensin-aldosterone system dysregulation observed with ageing. Fernandez-Vega et al. compared the safety and efficacy of candesartan with placebo in 618 post-menopausal hypertensive females. Systolic and diastolic BPs were significantly lower in the group treated with candesartan 16 mg/day compared with the placebo group (*n* = 321 vs. *n* = 265, difference in BP -19.9/ -11.5 mmHg, *p* < 0.01) and no serious adverse events were reported [76]. Consistent findings were also reported in a study conducted by Ikeda et al. on 69 hypertensive menopausal females. They found BP was significantly decreased in post-menopausal females with hypertension treated with 4 to 8 mg/day of candesartan following 12 months of treatment (157±21/85±11 vs. 141±18/77±12 mmHg, *p* < 0.001) [78].

#### Diuretics

Sodium retention and salt sensitivity are key mechanisms underlying elevated BP observed in ageing [57], and therefore increasing urinary sodium and water excretion is an essential part of the treatment of hypertension. The Females’s Health Initiative reported that diuretics provided better control of BP than any other class of drugs as monotherapy [79]. One trial compared the safety and efficacy of hydrochlorothiazide 25 mg and moexipril 15 mg in 97 post-menopausal females with hypertension [75]. After 12 weeks of treatment, there was no significant difference in BP lowering between the two groups (*n* = 48 vs. *n* = 49, -8.0±2.2/-10.0±1.3 mmHg vs. -15.5±2.1/ -11.8±1.1 mmHg, *p* > 0.05) [75]. However, the incidence of adverse events was significantly higher in the hydrochlorothiazide group, with headache and rhinitis being the most common events (54% vs. 40%, *p* < 0.05) [75]. In addition, the use of hydrochlorothiazide resulted in more metabolic effects, including hyperglycaemia, hyperuricaemia and hyperlipidaemia (*p* = 0.181, *p* = 0.004, *p* = 0.279 respectively) [75]. A significant association between diuretic use and total fractures was found in hypertensive post-menopausal females who had used cyclic diuretics for more than 3 years (HR 1.31, 95%CI 1.20–1.42). However, factors including previous medication history, gait and balance problems, and history of previous falls, were not considered in this study [75]. Rejnmark et al. conducted a study that directly compared the plasma calcium levels and urinary calcium excretion in response to 1 week of treatment of bendroflumethiazide (a thiazide diuretic) and bumetanide (loop diuretic) in 40 post-menopausal females with osteopenia [80]. It was found that urinary calcium excretion decreased in a dose-dependent manner with thiazide diuretics [80]. Urinary calcium excretion, plasma parathyroid hormone and calcitonin levels were increased in a dose-dependent manner with the use of loop diuretics, which may enhance bone loss and induce a negative calcium balance that may lead to bone loss [80]. Although future studies are needed to compare the long-term effects of using thiazide and loop diuretics on bone mineral density in post-menopausal females, the current study suggests that thiazide diuretics should be the preferred type of diuretic for this population.

#### Beta-blockers

Although generally not regarded as first line anti-hypertensive therapy, blocking beta-1 receptors can reduce BP by reducing cardiac contractility and renin release. This may be particularly of value in targeting the sympathetic activation observed in post-menopausal hypertension. A trial conducted by Kujala et al., involving 98 overweight, hypertensive post-menopausal females, showed a significant reduction in mean diastolic BP (*n* = 49 vs. *n* = 49, -12.9/-9.5 mmHg vs. -3.2/-6.2 mmHg, *p* < 0.001) and menopausal symptoms including hot flushes and palpitations after 8 weeks of 50 mg/day of atenolol treatment (43% vs. 27%, 33% vs. 27%, *p* = 0.0023 and *p* = 0.0012, respectively) [81]. Studies have suggested that beta-1 receptor blockers have comparable antihypertensive effect to other drugs in post-menopausal females [77, 82]. A study involving 120 post-menopausal females with mild to moderate hypertension comparing the antihypertensive effects of atenolol 50 mg and valsartan 80 mg found no significant difference between these two drugs after 16 weeks of treatment, apart from a significantly slower heart rate with atenolol (from 76.2 beats/min to 54.2 beats/min, *p* < 0.001 v baseline) [82].

#### Calcium Channel Blockers

Calcium channel blockers block the influx of calcium in the heart and smooth muscle of blood vessels, causing a decrease in cardiac contractility and reducing vasoconstriction to reduce BP. A study conducted by Agabiti-Rosei et al. comparing the ACE inhibitor moexipril vs. calcium channel blocker nitrendipine in 92 hypertensive post-menopausal females showed no significant difference in antihypertensive effect between the two groups after 8 weeks of treatment (*n* = 45 vs. *n* = 47, -21.2/-15.2 mmHg vs. -18.2/-13.6 mmHg, *p* > 0.05) [83]. However, moexipril was significantly better tolerated than nifedipine (22.2% vs. 53.2%, *p* < 0.05) [83]. Peripheral oedema was the main adverse effect in the nifedipine group (14.9%, *p* < 0.05) [83]. In addition, Hayoz et al. conducted a study investigating and comparing the effects of 10 mg of amlodipine and 320 mg of valsartan in 125 hypertensive post-menopausal females [84]. Following 38 weeks of treatment, both groups showed similar BP-lowering effects (*n* = 62 vs. *n* = 63, -25.2/-11.7 mmHg vs. -22.9/-10.9 mmHg, *p* > 0.05) [84]. Amlodipine was associated with a significantly higher incidence of peripheral oedema (77% vs. 14%, *p* < 0.001) [84]. Peripheral oedema is a common adverse effect of calcium channel blockers particularly in females. A few studies found prolonged use of calcium channel blockers was associated with a greater risk of both ductal and lobular breast cancer in post-menopausal females. A sub-analysis of the Cardiovascular Health Study (*n* = 3198 females aged ≥65 years) also showed a higher risk of invasive breast cancer (HR 2.57, 95%CI 1.47–4.49) with calcium channel blockers [85], but only 352 females were found to be exposed to calcium channel blockers for an average of 3.9 years. Although the link between breast cancer and calcium channel blockers is unclear, one hypothesis is due to accumulation of calcium. Calcium plays an important role in cell differentiation and in the apoptotic process. The use of calcium channel blocker may act as a tumour promotor by interfering with the programmed death of DNA-damaged cells [86, 87]. More studies are required to investigate this relationship.

#### Hormonal Replacement Therapy

While oestrogen deficiency is a key mechanism predisposing to hypertension via its effects of multiple pathways, the antihypertensive effect of hormone replacement therapy remains controversial [95]. On the one hand, some studies of hormone replacement therapy show that the therapy did not appear to lower BP [88–91]. For example, a Japanese study showed hormonal replacement therapy (0.625 mg of conjugated equine oestrogen combined with 2.5 mg of medroxyprogesterone) increases the incidence of hypertension in post-menopausal females after a median of 5.6 years of therapy compared to placebo (*n* = 5994 vs. *n* = 5679, HR 1.18, 95%CI 1.90–1.27) [92]. In another study, Jaszcz et al. reported no effect of hormone replacement therapy (transdermal hormone substitution with 17β-oestradiol and norethisterone acetate) on arterial BP in hypertensive post-menopausal females compared to the control group (*n* = 40 vs. *n* = 36, -2.75±4.0/-8.4±1.0 mmHg vs. -4.9±7.6/-8.8±2.9 mmHg, *p* > 0.05) [89]. Scuteri et al. reported that post-menopausal females taking hormone replacement therapy for an average of 5.7 years had comparable average increases of both systolic and diastolic BP between the hormone replacement therapy group (oral or transdermal oestrogen and progestin) and control group (*n* = 149 vs. *n* = 77, 38.3/12.0 mmHg vs. 37.4/9.7 mmHg, *p* > 0.2) [93]. Higashi et al. reported the benefit of oestrogen replacement therapy on endothelial function in hypertensive post-menopausal females (*p* < 0.01), but direct effects and evidence on BP were lacking [61]. Another trial suggested that systolic BP was lower in females on oestrogen replacement therapy, but not on progestogen-containing hormone replacement therapy, which might partially explain the ambiguous results of progestogen-containing hormone replacement therapy on BP [94]. However, a recent small study found that both oestrogen therapy and oestrogen + progestin therapy reduced BP in hypertensive post-menopausal females with no significant difference in BP reduction between the two groups (*n* = 19 vs. n-17, -1.0±13.4/-2.5±7.9 mmHg vs. -1.0±5.4/-1.3±5.4 mmHg, *p* = 0.85) [95]. Overall, there are currently inconsistent findings regarding the effect of hormone replacement therapy on BP. In addition to this, the optimal route of administration, start time and duration are controversial [43]. 

Drospirenone is a novel progestogen with aldosterone receptor antagonist activity. By blocking aldosterone receptors, drospirenone reduces salt and water retention, thereby reducing blood volume and BP. A new hormone therapy that combines drospirenone with 17β-estradiol (DRSP/E2) has been proven to have a promising antihypertensive effect. A study in 213 hypertensive post-menopausal females found that after 12 weeks of treatment, DRSP/E2 (3 mg DRSP/1 mg E2) significantly reduced BP compared to placebo (*n* = 102 vs. *n* = 111, -14.1/-7.9 mmHg vs. -7.1/-4.3 mmHg, *p* < 0.0001) [96]. Due to the anti-mineralocorticoid effects of DRSP, changes in serum potassium remain a concern. However, this study found no significant difference in potassium levels between the two groups (0.24±0.38 meq/L vs. 0.16±0.43 meq/L, *p* = 0.18), and no other adverse events were reported during the treatment [96]. Preston et al. conducted another study that investigated the effect of a combination of DRSP/E2 and hydrochlorothiazide (3 mg DRSP + 25 mg HCTZ) in 36 hypertensive post-menopausal females following 56 days of treatment. This study showed this combination significantly lowered BP (*n* = 18 in both groups; -7.2/-4.5 mmHg, 95%CI -6.9–2.1 mmHg, *p* < 0.05) with minimal effect on serum potassium (0.27 vs. 0.06 meq/L, *p* = 0.0059) [97]. Consistent findings were also reported in a meta-analysis that included 5 clinical trials of DRSP/E2 in hypertensive post-menopausal females [98]. Although current trials have shown positive clinical effects, future large-scale studies are needed to investigate its long-term effects.

#### Blood Pressure Control in Post-menopausal Females

Hypertensive females aged 70–79 years are as likely to be on treatment as younger hypertensive females. However, BP control rates are still lower in older hypertensive females. A national study conducted by the Females Health Initiative across the United States evaluated the pattern of treatment and adequacy of BP control, involving 98,705 hypertensive post-menopausal females aged from 50 to 79 years old [79]. Although 64% of hypertensive females in the study were treated with antihypertensive medication, BP was only controlled in about a third of them. Apart from changes in physiology due to ageing and hormonal effects, other factors could contribute to females responding less to antihypertensive regimens. Firstly, the reduced rate of control might be related to the differences in the pharmacokinetic profile of drugs in females. Compared to men, females have a higher gastric pH, which slows gastric emptying. These differences can reduce the bioavailability of drugs that require an acidic environment for absorption. Compared to men, females’ drug metabolism is characterised by increased activity of the CYP450 enzyme system [99, 100]. These enzymes are involved in the metabolism of about 50% of drugs. Increased metabolism of drugs can lead to decreased plasma concentration, therefore, reducing the therapeutic effect of drugs. Secondly, some antihypertensives have gender-specific adverse effects, with females reporting adverse effects more frequently than males. Cough is three times more common in females than males following the treatment of ACE inhibitors [101]. Diuretic-induced hypokalaemia and hyponatremia were reported to be more frequent in females than in men [102–104]. A high incidence of peripheral oedema with dihydropyridine calcium channel blockers has also been observed in females than in males [105–107]. These may lead to discontinuation and non-compliance with treatment in females [108].

### Prognosis

Studies investigating the long-term prognosis of females with post-menopausal hypertension are limited. A few studies have found females with post-menopausal hypertension are at increased risk of adverse cardiovascular outcomes including myocardial infarction, heart failure, coronary dissection [109]. There is also an increased association with cognitive decline and dementia [110, 111].

There is emerging evidence that cardiovascular risk increases at a lower BP threshold in females than in males, including risk for myocardial infarction, heart failure and stroke [5, 109, 112]. The INTERHEART study was a case-control study performed in 52 countries which demonstrated that hypertension was associated with greater risk of myocardial infarction in older females than age-matched males [113]. This is consistent with the Tromso Study, which also found that higher BP was a stronger risk factor for myocardial infarction in females than males [114]. Whether this is related to underestimation of cuff systolic BP to invasive aortic systolic BP in females compared with males is unknown [115].

Non-dipping nocturnal BP in hypertensive females was associated with increased risk of cardiovascular disease [116]. It has also been observed that females have greater BP variability in ambulatory BP readings [117], which is a marker of increased risk of cardiovascular events including coronary heart disease and stroke [118].

Hypertension increases the risk of CCF in females by 1.5 times compared to age- and risk factor-matched men [119]. This may be due to gender-related physiological differences in cardiac and vascular changes in response to chronic hypertension. Post-menopausal females with hypertension have a much higher incidence of left ventricular hypertrophy than both age-matched males and premenopausal females (*p* = 0.001), and therefore an increased incidence of diastolic dysfunction leading to CCF (*p* = 0.001) [120]. Left ventricular hypertrophy in hypertension is associated with reduced myocardial function as determined by fractional mid wall shortening or global longitudinal strain [120]. This may account for the higher prevalence of heart failure with preserved ejection fraction observed in females than in males. A meta-analysis pooling data from 3 prospective studies that included 3568 heart failure events, showed females who experienced early menopause had a significantly higher risk of post-menopausal heart failure (HR 1.33; 95%CI 1.53) [86].

Post-menopausal hypertension may also be associated with increased risk of coronary artery dissection. A prospective study involving 327 patients with spontaneous coronary artery dissection discovered that a large proportion of these patients had a history of hypertension [87]. A similar finding was found in a study conducted by Chang et al. [121]. These may suggest an association between post-menopausal hypertension and the development of coronary artery dissection, but future large-scale studies are needed. Although the mechanism is not clear, one hypothesis is that hypertension can induce arterial shear stress, ultimately leading to coronary artery dissection [87].

There is also increasing evidence suggesting an association between cognitive decline with midlife hypertension in females. Observational data demonstrated that mid-adulthood hypertension was associated with a 65% (95%CI 1.25–2.18) increased risk of dementia among females but not men [110]. Similarly, another population-based study demonstrated that incident midlife hypertension was associated with greater memory decline in females but not men [111]. Oestrogen protects the brain through its antioxidant properties and oestrogen receptor-mediated genomic effects [122]. Studies using post-menopausal mouse models have found that decreased systemic and cerebral oestrogen levels downregulate oestrogen receptor expression in the cortex and hippocampus and reduce myelin basic protein expression in the corpus callosum [123, 124]. These factors together may increase the risk of dementia in women with post-menopausal hypertension.

### Future Directions in the Management of Hypertension in Females

**Fig. 3:**
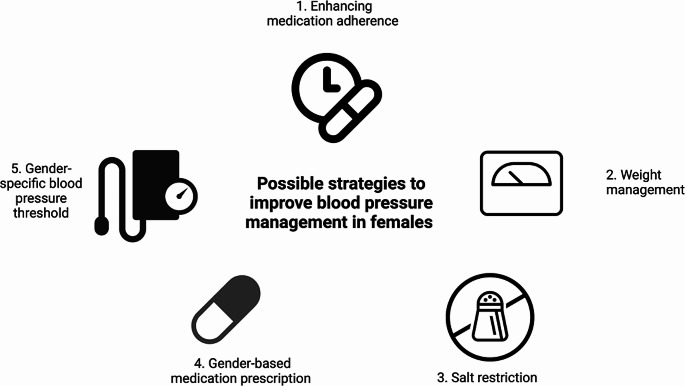
Potential strategies to reduce blood pressure in women

Given the disparities in treatment and poor prognostic outcomes in females with high BP compared to aged matched men, there need to be greater understanding of the underlying mechanisms for BP-mediated organ damage development in females. New strategies for treatment of high BP in females are needed, including identification of high-risk phenotypes and progression to cardiovascular diseases. In the absence of clear sex-specific treatment strategies, there needs to be greater emphasis on addressing the poor adherence noted in females and targeting risk factors associated with high BP in females including optimal weight management and salt restriction (Fig. 3). Use of low-dose single pill combination anti-hypertensive therapy may also be of value to improve adherence and minimise adverse effects [125]. Future clinical studies should explore whether using sex-specific BP threshold values and treatment targets in hypertension may improve cardiovascular disease prevention.

## Conclusion

Hypertension is a key risk factor for cardiovascular disease in females, who appear to develop cardiovascular disease at lower BP thresholds and be more vulnerable to treatment-related adverse effects than men. Sex-specific risk factors, including pregnancy and menopause, make females more likely to have more adverse cardiovascular outcomes than age-matched men. Despite scientific advances, gaps in management outcomes persist between the two sexes. Current high BP treatment guidelines and recommendations are similar for both sexes, without addressing sex-specific factors. BP trials continue to have an inadequate representation of females. Therefore, future investigations into ideal diagnostic thresholds, BP control targets and treatment regimens are needed in females.

## Data Availability

No datasets were generated or analysed during the current study.
